# *Boswellia serrata* Preserves Intestinal Epithelial Barrier from Oxidative and Inflammatory Damage

**DOI:** 10.1371/journal.pone.0125375

**Published:** 2015-05-08

**Authors:** Daniela Catanzaro, Serena Rancan, Genny Orso, Stefano Dall’Acqua, Paola Brun, Maria Cecilia Giron, Maria Carrara, Ignazio Castagliuolo, Eugenio Ragazzi, Laura Caparrotta, Monica Montopoli

**Affiliations:** 1 Department of Pharmaceutical and Pharmacological Sciences, University of Padova, Largo E. Meneghetti 2, 35131, Padova, Italy; 2 IRCCS "E. Medea," Conegliano, Italy; 3 Department of Molecular Medicine, University of Padova, via Gabelli 63, 35121, Padova, Italy; Hungarian Academy of Sciences, HUNGARY

## Abstract

Aminosalicylates, corticosteroids and immunosuppressants are currently the therapeutic choices in inflammatory bowel diseases (IBD), however, with limited remission and often serious side effects. Meanwhile complementary and alternative medicine (CAM) use is increasing, particularly herbal medicine. *Boswellia serrata* is a traditional Ayurvedic remedy with anti-inflammatory properties, of interest for its usefulness in IBDs. The mechanism of this pharmacological potential of *Boswellia serrata* was investigated in colonic epithelial cell monolayers exposed to H_2_O_2_ or INF-γ+TNF-α, chosen as *in vitro* experimental model of intestinal inflammation. The barrier function was evaluated by the transepithelial electrical resistance (TEER) and paracellular permeability assay, and by the tight junction proteins (zonula occludens-1, ZO-1 and occludin) immunofluorescence. The expression of phosphorylated NF-κB and reactive oxygen species (ROS) generation were determined by immunoblot and cytofluorimetric assay, respectively. *Boswellia serrata* oleo-gum extract (BSE) and its pure derivative acetyl-11-keto-β-boswellic acid (AKBA), were tested at 0.1-10 μg/ml and 0.027μg/ml, respectively. BSE and AKBA safety was demonstrated by no alteration of intestinal cell viability and barrier function and integrity biomarkers. H_2_O_2_ or INF-γ+TNF-α treatment of Caco-2 cell monolayers significantly reduced TEER, increased paracellular permeability and caused the disassembly of tight junction proteins occludin and ZO-1. BSE and AKBA pretreatment significantly prevented functional and morphological alterations and also the NF-κB phosphorylation induced by the inflammatory stimuli. At the same concentrations BSE and AKBA counteracted the increase of ROS caused by H_2_O_2_ exposure. Data showed the positive correlation of the antioxidant activity with the mechanism involved in the physiologic maintenance of the integrity and function of the intestinal epithelium. This study elucidates the pharmacological mechanisms mediated by BSE, in protecting intestinal epithelial barrier from inflammatory damage and supports its use as safe adjuvant in patients affected by IBD.

## Introduction

Inflammatory bowel diseases (IBD), mainly Crohn's disease and ulcerative colitis, affect more than 4 million people in the world, with a clinical onset typically between 15 and 45 years, becoming a clinical challenge in terms of treatments [1, 2]. Microbiota, genetic, and immunological factors are involved in the etiopathogenesis of IBDs and the intestinal barrier dysfunction is the main feature causing leak flux diarrhea and facilitating uptake of noxious antigens [3–5]. Aminosalicylates and glucocorticosteroids are current therapy to control inflammation and symptoms. However, in severe cases immunosuppressive drugs, such as azathioprine, 6-mercaptopurine, cyclosporine or anti-TNF α monoclonal antibody are prescribed regardless the adverse complications and the limited remission [6, 7]. Thus, complementary and alternative medicine (CAM) use is increasing, particularly herbal medicine, as safe anti-oxidant/anti-inflammatory therapeutic option in IBD patients [8, 9]. Indeed, from one hand, oxidative injury is relevant in the initiation and progression of barrier dysfunction, due to the enhanced production of reactive oxygen species (ROS) and reactive nitrogen species (RNS) in intestinal mucosa, mainly triggered by neutrophils and activated leukocytes, causing lipid peroxidation, protein modification and pro-inflammatory cytokines production [10]. On the other hand, the release of TNF-α and INF-γ induces intestinal damage in IBD, such as changes in tight junction (TJ) structures, apoptosis and enhanced bacterial translocation [11], that can be reproduced in cell and animal experimental models.

*Boswellia serrata* oleo-gum resin, a traditional Ayurvedic remedy for inflammatory diseases also known as *Salai Guggal*, *Indian olibanu*, Indian frankincense, is obtained from *Boswellia* genus (Family Burseraceae). *Boswellia* antioxidant/antinflammatory properties have been studied for the pharmacological potential in arthritis, asthma, colitis and cancer [12–16]. The phytochemical content of *Boswellia serrata* oleo-gum resin is dependent on the botanical origin and consists of 30–60% triterpenes (such as α- and β-boswellic acids, lupeolic acid), 5–10% essential oils, and polysaccharides. The 11-keto-β-boswellic acid (KBA) and acetyl-11-keto-β-boswellic acid (AKBA) have been considered as the main active derivatives [17], and several mechanisms of action have been demonstrated: inhibition of 5-lipoxygenase (5-LO), effects on immune system, such as decreased cytokines (interleukins and TNF-α) levels and diminished complement system and leukocyte elastase activities, reduction of ROS formation and P-selectin-mediated recruitment of inflammatory cells [18–20]. However, other components of the oleo-gum resin, such as β-boswellic acid, have been suggested as anti-inflammatory agents, acting through inhibition of serine protease cathepsin G and microsomal prostaglandin E synthase [17]. Since the therapeutic potential of *Boswellia* in IBD is still under debate, in this study *Boswellia serrata* oleo-gum resin extract (BSE) and its derivative AKBA were evaluated in Caco-2 cell monolayer exposed to H_2_O_2_ or to INF-γ+TNF-α, chosen as experimental model of endogenous inflammatory stimuli [21]. Functional, morphological and molecular biomarkers of intestinal barrier integrity were investigated.

## Material and Methods

### HPLC-MS and HPLC-DAD-ELSD analyses

Qualitative and quantitative constituents of BSE (resin dry extract water/ethanol 60/40 V/V kindly provided by EOS, Treviso, Italy) were performed by high-performance liquid chromatography-tandem mass spectrometry (HPLC-MS) and high-performance liquid chromatography coupled with a diode array detector and an evaporative light scattering detector (HPLC-DAD-ELSD). BSE (50 mg) was dissolved in 15 ml of methanol using an ultrasound bath for 10 minutes. After centrifugation (5 min at 3000 rpm), the solid residue was extracted again with 10 ml of methanol in ultrasound bath for 10 min, centrifuged, and the supernatant collected to 100 ml with the same solvent. A portion of the solution was filtered through 0.45 μm membrane and used for HPLC analysis.

For HPLC-MS analysis a Varian 212 binary chromatograph equipped with 500MS ion trap and Prostar 430 autosampler was used (Varian Inc., USA). For the HPLC-DAD-ELSD analysis an Agilent 1100 Series chromatograph with 1100 Diode Array detector and Sedex LX 60 Evaporative Light Scattering Detector (ELSD) was used. As stationary phase an Agilent Eclipse XDB-C8 2.1 x 150 mm, 3.5 μm (Agilent Tecnologies, USA) was used. The mobile phase was composed of methanol (A) and water with 0.1% formic acid (B) and the gradient phases were: 70% A to 100% A in 10 minutes, then isocratic phase until 30 minutes. Re-equilibration time was from 31 to 44 minutes. The flow was 200 μl/min and the injected volume was 10 μl. The same column and gradient were used both in the HPLC-MS and HPLC-DAD-ELSD systems. The MS parameter were: positive ion mode, 84 Volt capillary voltage, 50–2000 m/z of spectral range, RF loading 70%, nebulizing gas Nitrogen pressure 25 psi, drying gas temperature 350°C and drying gas pressure 15 psi, spray shield 600 volt. Triterpenes were identified on the basis of their MS spectra and comparison with the literature. For the quantitative determination, 5–150 μg/mL solutions of 11-acetyl-α-keto boswellic acid (AKBA, Extrasynthese, France) were prepared. Calibration curve was obtained measuring the peak area at 260 nm with the DAD detector. The equation of the curve was y = 0.0187x+ 0.5218 and R^2^ was 0.9999. The ELSD was used as detector of signals also from triterpene lacking the chromophore groups. A calibration curve was obtained (y = 0.0232 x^2^ + 0.23 x+ 0.0022; R^2^ = 0.9998) also with the ELSD and the total amount of triterpene was calculated using AKBA as reference compound.

### Intestinal cell monolayer preparation and treatment

LoVo and HT29 cells were purchased from American Type Culture Collection (Manassas, VA, USA) and grown in Roswell Park Memorial Institute medium (RPMI 1640) whereas Caco-2 cells, obtained from American Type Culture Collection, were grown in high-glucose Dulbecco’s modified Eagle’s media (DMEM). grown in high-glucose Dulbecco’s modified Eagle’s media (DMEM). Medium was supplemented with 10% Fetal Bovine Serum (FBS), 2% L-glutamine and 1% penicillin/streptomycin. Cells were maintained under a humidified atmosphere of 5% CO_2_ in air, incubator at 37°C. Experimental inflammatory condition in Caco-2 cell monolayers was induced by the exposure for different times according to the assays, to 500 μM H_2_O_2_ or to 2.5 ng/ml recombinant human IFN-γ for 3 hours and then 10 ng/ml TNF-α were added. Twenty-four hours pre-treatment with BSE (0.1–1 μg/ml) or AKBA (0.027 μg/ml) was applied before the inflammatory stimuli. Reagents for cell cultures were from Cambrex-Lonza (Basel, Switzerland), whereas INF-γ and TNF-α were from R&D Systems (Minneapolis, USA).

### Cell viability assay

Cell viability was determined by the 3-(4,5-dimethyl- thiazole-2-yl)-2,5-diphenyltetrazolium bromide (MTT) assay, as previously described [22]. Briefly, LoVo, HT-29 and Caco-2 cells were seeded in 96-multiwells culture plates and treated with BSE (0.1–10 μg/ml) or AKBA (0.027 μg/ml) for 24, 48 or72 hours. The formazan absorbance was measured at 570 nm, using a Multilabel Plate Reader VICTOR X3 (PerkinElmer).

### ROS fluorescence assay

ROS were quantified using 2′,7′-dichlorofluorescin-diacetate (H_2_-DCF-DA, Sigma-Aldrich), as previously described [23]. Upon cleavage of the acetate groups by intracellular esterase and oxidation, the H_2-_DCF-DA is converted to the fluorescent 2',7'-dichlorofluorescein (DCF). Briefly, the intestinal cells (5×10^3^) were seeded into 96-well plates and allowed to adhere overnight. ROS level was measured after the exposure to BSE or AKBA for 24 hours, in the absence or presence of 500 μM H_2_O_2_, and subsequent addition of 10 μM H_2_-DCF-DA, further incubation for 30 min at 37°C and washing with phosphate-buffered saline (PBS). DCF fluorescence intensity was measured at excitation 485 nm—emission 535 nm, using a Multilabel Plate Reader VICTOR X3 (PerkinElmer). Fold increase in ROS production was calculated using the equation: (F_treatment_—F_blank_)/(F_control_—F_blank_), where F is the fluorescence reading.

### Trans-epithelial electrical resistance (TEER) assay

Caco-2 cell cultures were performed essentially as previously described [24]. Briefly, the cells were seeded on Transwell polyester membrane cell culture inserts (transparent PET membrane: 1.0 cm^2^ growth surface area, 0.4 μm pore size; BD Falcon) in 24-well plates and incubated with the appropriate culture media at 37°C in a humidified atmosphere and 5% CO_2_. Culture media were replaced every day until confluent monolayer was obtained. The TEER assay was performed in HBBS (Hanks’ Balanced Salt solution, Cambrex-Lonza, Basel, Switzerland) after an equilibration period at room temperature. Treatments were added to the basal chamber without manipulating the apical media. Millicell ERS meter, Millipore Corporation (Bedford, MA) connected to a pair of chopstick electrodes were inserted in the donor and receiver chambers and the 24 hours-time course of TEER variation was recorded. TEER was expressed as percentage of resistance, normalized to initial value.

### Paracellular permeability assay

Sodium fluorescein flux across Caco-2 cell monolayers was used as a measure of the paracellular permeability. Caco-2 cell culture were used essentially as previously described [25]. Briefly, the cells were seeded on Transwell polyester membrane cell culture inserts (transparent PET membrane: 1.0 cm^2^ growth surface area, 0.4 μm pore size; BD Falcon) in a 24-well plate with the appropriate medium. The cells were incubated at 37°C in a humidified atmosphere of 5% CO_2_ and confluent monolayers were obtained within 20–21 days. Culture media were replaced every day. 200 μl of HBBS (Hanks’ Balanced Salt solution, Cambrex-Lonza, Basel, Switzerland) containing 1 mg/ml sodium fluorescein were added to the apical side of each monolayer chamber. At the end of experimental protocols, the amount of fluorescein permeation to the basolateral side of the chamber was measured using a Multilabel Plate Reader VICTOR X3 (PerkinElmer) at excitation 480 nm—emission 530 nm.

### Immunofluorescence microscopy

At the end of the experimental protocols cells were collected by trypsinization and centrifugation, washed with PBS, seeded on glass coverslips in 6-well plates and cultured until reached approximately 30% confluence. For detection of ZO-1 and occludin TJ proteins, cells were fixed with 4% formaldehyde, permeabilized with 0.1% Triton X-100 in PBS and stained with a mouse monoclonal anti-occludin or with a rabbit polyclonal anti-ZO-1 (Invitrogen Life Technologies, Milan, Italy). After PBS wash, they were incubated with secondary antibodies/fluorescein isothiocyanate (Alexa Fluor 488 anti-mouse or Alexa Fluor 536 anti-rabbit immunoglobulin G, Molecular Probes, Invitrogen, Milan, Italy) for 1 hour at room temperature. The coverslips were mounted on glass slides by using Mowiol 40–88 (Sigma, St Louis, MO). Images were acquired through a ×60 CFI Plan Apochromat Nikon objectives with a Nikon C1 confocal microscope and finally analysed using NIS Elements software (Nikon Instruments, Florence, Italy), NIH Image J and Adobe Photoshop CS4 version 11.0.2.

### NF-κB immunoblot and chemiluminescence analysis

At the end of the experimental protocol Caco-2 cell monolayers were washed twice in ice-cold PBS and then lysed (45 min on ice) using nondenaturing RIPA buffer (150 mM NaCl, 50 mM Tris-HCl, 0.25% sodium deoxycholate, 0.1% Nonidet P-40, 100 μM NaVO4, 1mM NaF, 1 mM phenylmethylsulfonyl fluoride, 10 μg/ml aprotinin, 10 μg/ml leupeptin from Sigma, St Louis, MO, USA). Particulate material was removed by centrifugation (15000 x *g* for 5 min at 4°C). Supernatants were collected and protein concentrations were determined using the bicinchoninic acid method (Pierce, Rockford, IL, USA). Twenty-five μg of total proteins were fractionated through a 7.5% p/v SDS-PAGE gel and then transferred and immobilized onto a nitrocellulose membrane. Membranes were blocked for 20 min at room temperature in 5% skim milk (Oncogene, Cambridge, MA, USA) in PBS containing 0.05% v/v Tween-20. After washes, membranes were incubated for 16 hours at 4°C with mouse anti-phospho-NF-κB p65 antibody (7F1, Cell Signaling, Danvers, MA, USA). Bound antibody was detected by incubation with HRP-conjugated goat anti-mouse IgG antibody. Bands were visualized using enhanced chemiluminescence (Millipore, Billerica, MA, USA). Images were captured using a Hyper Film MP (GE Healthcare, Milan, Italy) and densitometry was performed using NIH Image J software.

### Statistical Analysis

The statistical analysis was performed using GraphPad Prism version 3 for Windows (GraphPad Software, San Diego, CA, USA). Unless stated otherwise, results are presented as mean±SEM. The unpaired *t*-test was used to compare ROS intracellular level, paracellular permeability and NF-κB phosphorylation in different experimental conditions. *P* values <0.05 were considered statistically significant. ANOVA followed by a *post-hoc* test (Dunnett's test or Tukey’s test for multiple comparisons) were used to statistically evaluate trans-epithelial electrical resistance (TEER) variations during 24 hours of different treatments.

## Results

### HPLC-MS and HPLC-DAD-ELSD quali-quantitative analysis of BSE

The anti-inflammatory properties of *Boswellia serrata* are also attributed to 11-keto-β-boswellic acid (KBA) and 3-acetyl-11-keto-β-boswellic acid (AKBA), even if other boswellic acids, such as β-boswellic acid (βBA) might be efficacious. Therefore, the first step of our study was to measure BSE triterpene content by HPLC-MS and HPLC-DAD-ELSD procedures. Results, summarized in Table 1, show that triterpene derivatives composed the 39% of total BSE where KBA and AKBA, in particular, resulted to be at the percentage of 5.02% and 2.71%, respectively.

**Table 1:** Main triterpene content in the BSE: retention time (Rt), adduction ion ([M + H]^+^, formed by the interaction of the molecule with a proton), and percentage amount.

Rt	Compound	[M+H]^+^	% in BSE
10.0	11-keto-β-boswellic acid (KBA)	471	5.02± 0.09
11.0	3-O-acetyl-11-keto-β-boswellic acid (AKBA)	513	2.71 ± 0.09
11.3	9,11-dehydro-α-boswellic acid	455	4.40 ± 0.04
11.6	β-amirin	427	4.04 ± 0.06
12.3	9,11-dehydro-β-boswellic acid	455	4.68 ± 0.03
12.5	α-boswellic acid	457	1.40 ± 0.05
12.7	β-boswellic acid	457	0.94 ± 0.04
12.8	lupeolic acid	455	2.84 ± 0.06
11.8	3-O-acetyl-9,11-dehydro-α-boswellic acid	497	2.42 ± 0.02
12.1	3-O-acetyl-9,11-dehydro-β-boswellic acid	497	0.67 ± 0.05
12.2	ursanic acid	499	0.87 ± 0.03
12.9	3-O-acetyl-α-boswellic acid	499	1.77 ± 0.05
13.0	3-O-acetyl-β-boswellic acid	499	1.61 ± 0.02

Data are the mean ± SD of 3 determinations.

### BSE and AKBA do not affect intestinal cell viability

**Fig 1:**
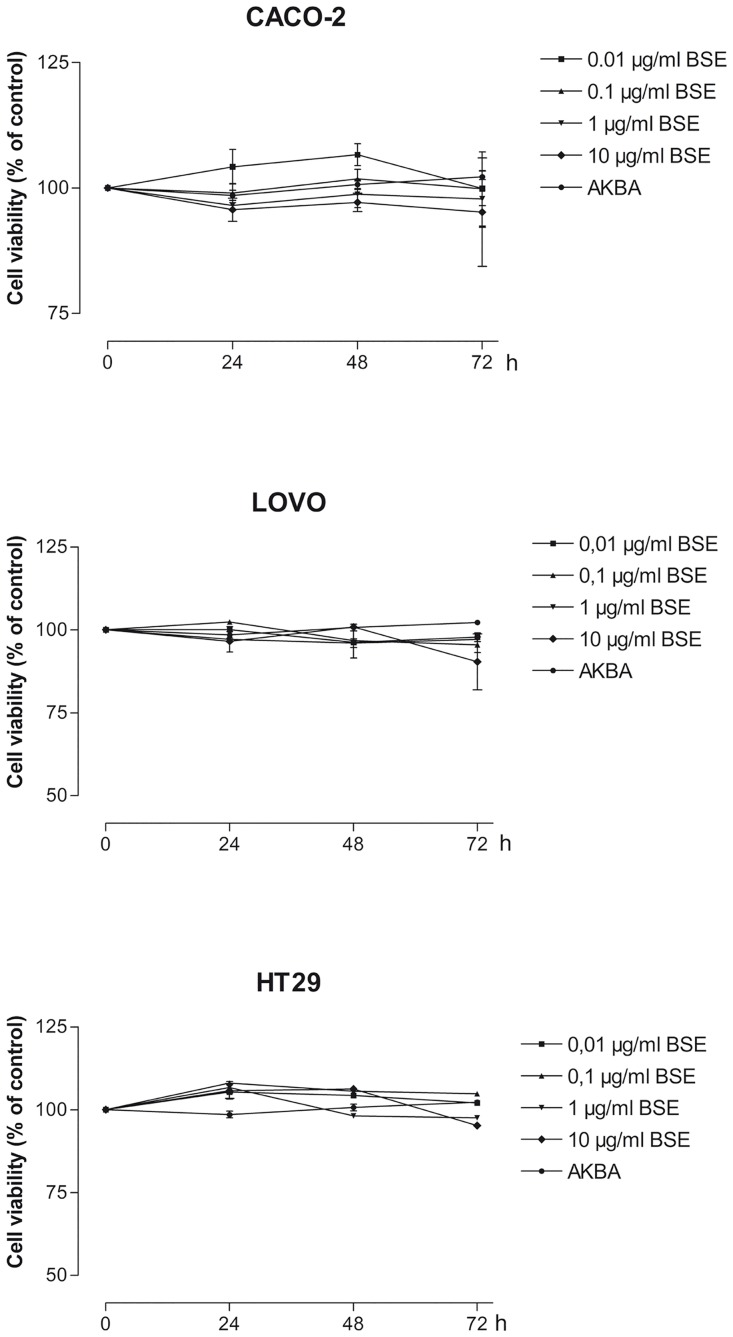
Effect of BSE and AKBA on cell viability measured by MTT assay in Caco-2, LoVo and HT-29 intestinal epithelial cell lines. Results are mean ±SEM of n = 4 experiments and are expressed as Absorbance Units and normalized to values in untreated cells (100%).

Caco-2, LoVo and HT-29 intestinal epithelial cell monolayers were treated for 24, 48 and 72 hours with BSE (0.01–10 μg/ml) or with AKBA (0.027μg/ml, corresponding to 2.7% of AKBA present in 1 μg/ml of BSE). Cell viability was assessed by MTT assay and Fig 1 shows that both BSE and AKBA did not affect the viability of the three types of intestinal cell lines.

### BSE and AKBA prevent the dysfunction of trans-epithelial electrical resistance and paracellular permeability caused by oxidative/inflammatory stimuli

**Fig 2:**
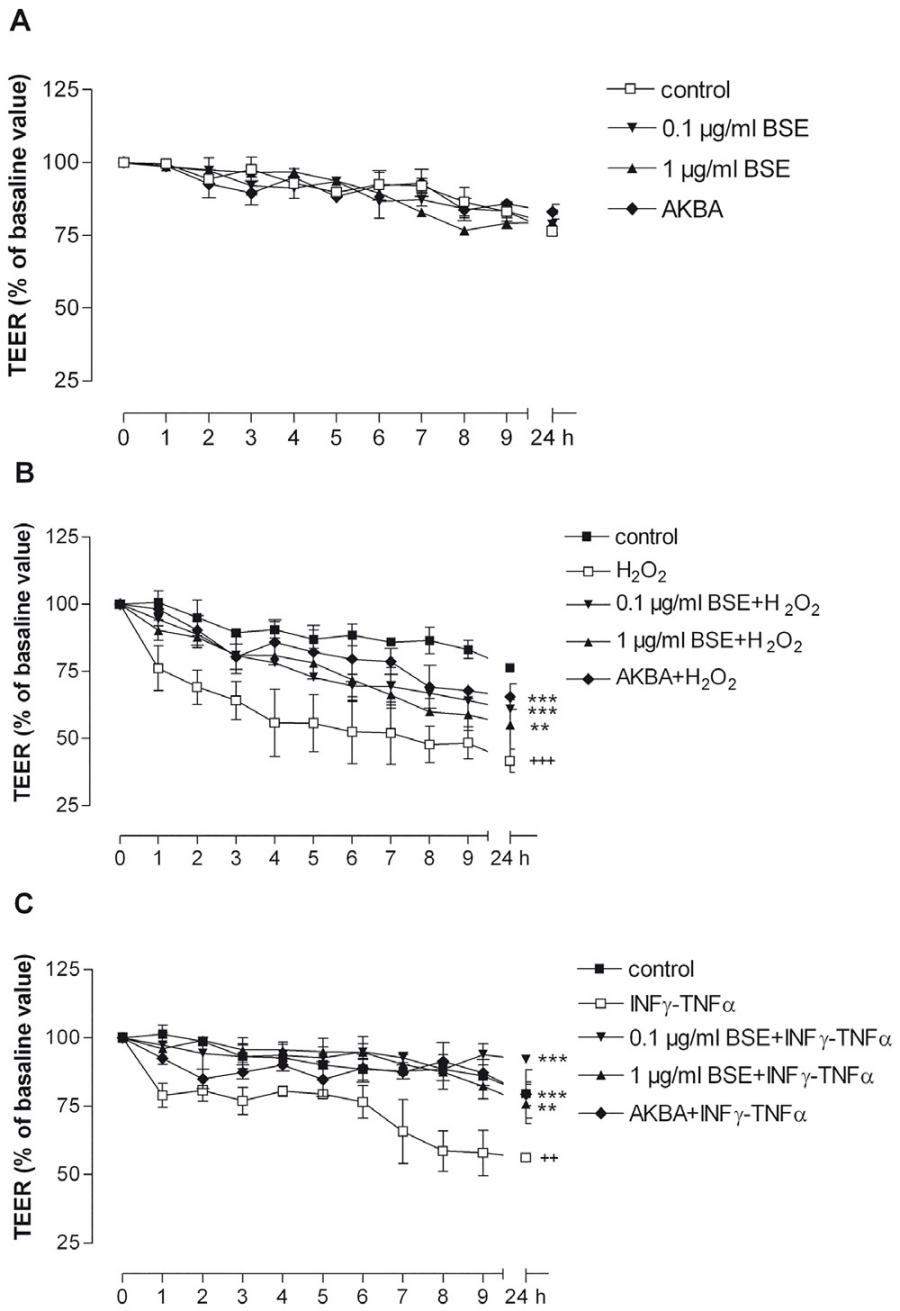
Effect of BSE and AKBA on transepithelial electrical resistance (TEER) in Caco-2 cell monolayers. A) BSE and AKBA; B) BSE and AKBA with H_2_O_2_ (^+++^ *p*<0.001 H_2_O_2_ *vs* control. ***p<0.001 BSE and AKBA vs H_2_O_2_ **p<0.01 BSE and AKBA *vs* H_2_O_2_); C) BSE or AKBA with INFγ+TNFα (^++^ *p*<0.01 INF-γ+TNF-α *vs* control ***p<0.001 BSE and AKBA *vs* INF-γ+TNF-α **p<0.01 BSE and AKBA *vs* INF-γ+TNF-α). Data are expressed as mean ± SEM percentage of baseline TEER value of n = 3 experiments.

Since uncontrolled or dysregulated gut epithelial permeability in overactive mucosal immune response and chronic intestinal inflammation has been proposed as primary defects in IBD, TEER and paracellular permeability are considered specific and sensitive biomarkers of the intestinal barrier integrity and function [24, 25]. Thus, the effect of BSE (0.1μg/ml, 1μg/ml) or AKBA (0.027 μg/ml) was measured on TEER and permeability in Caco-2 cell monolayer in basal condition and after exposure to H_2_O_2_ or to INF-γ+TNF-α (Fig 2).

Twenty-four hours of BSE or AKBA treatment did not cause any alteration of basal TEER (Fig 2A) whereas 24 hours-exposure to 500 μM H_2_O_2_ determined a time-dependent reduction of more than 50% of TEER T_0_ value (Fig 2B). This effect was significantly prevented by BSE or AKBA pretreatment (*p* = 0.003 and *p* = 0.0043, respectively, ANOVA followed by Dunnett’s test towards H_2_O_2_). INF-γ + TNF-α treatment also significantly decreased TER T_0_ value (*p* = 0.0003; Fig 2C) and BSE or AKBA prevented also in this case the intestinal cell polarity reduction (*p* = 0.02; Fig 2C).

**Fig 3:**
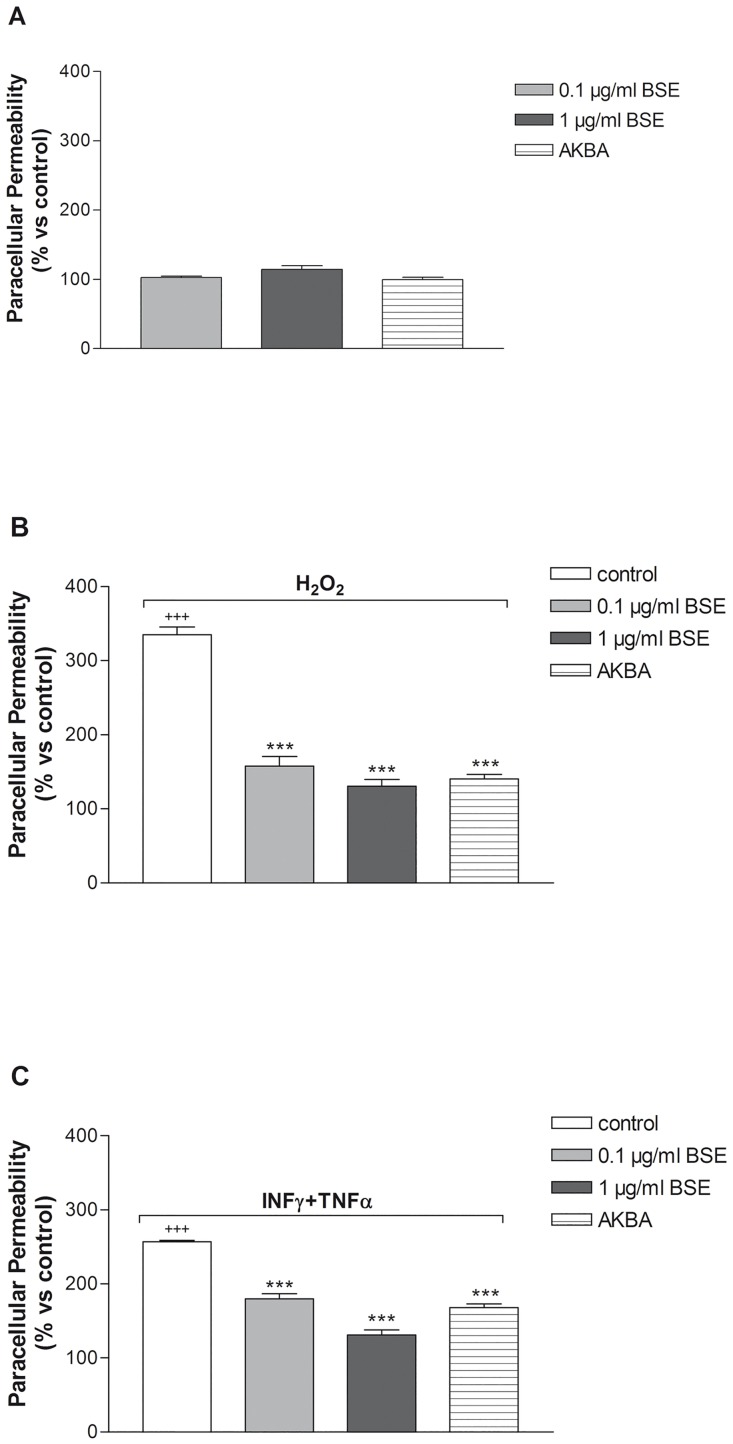
Effect of BSE and AKBA on Caco-2 cell monolayers paracellular permeability, measured by sodium fluorescein assay. A) basal condition. B) BSE and AKBA + H_2_O_2_ (^+++^ *p*<0.001 H_2_O_2_ *vs* control ***p<0.001 BSE and AKBA *vs* H_2_O_2_ **p<0.01 BSE and AKBA *vs* H_2_O_2_). C) BSE and AKBA + INF-γ+TNF-α. (^+++^p<0.001 INFγ+TNFα *vs* control ^+^ *p*<0.05 INF-γ+TNF-α *vs* control ***p<0.001 BSE and AKBA *vs* INF-γ+TNF-α). Data are shown as mean ± SEM percentage of basal fluorescent intensity (FI) of n = 3 experiments.

Sodium fluorescein is a validated biomarker of leakage used in the paracellular permeability assay [26]. The assay was applied to evaluate the effect of inflammatory stimuli in presence of absence of BSE and AKBA treatment in Caco-2 cell monolayer (Fig 3). BSE and AKBA did not influence the cell permeability in basal condition (Fig 3A). However, H_2_O_2_ and INF-γ+TNF-α increased basal values more than three-fold (*p* <0.0001) and more than two fold (*p* = 0.005), respectively (Fig 3B and 3C). When Caco-2 cell monolayers were pretreated with BSE and AKBA, the alteration of paracellular permeability induced by inflammatory stimuli was significantly counteracted (*p* <0.0001).

### BSE and AKBA prevent TJ disruption induced by oxidative and inflammatory stimuli

**Fig 4:**
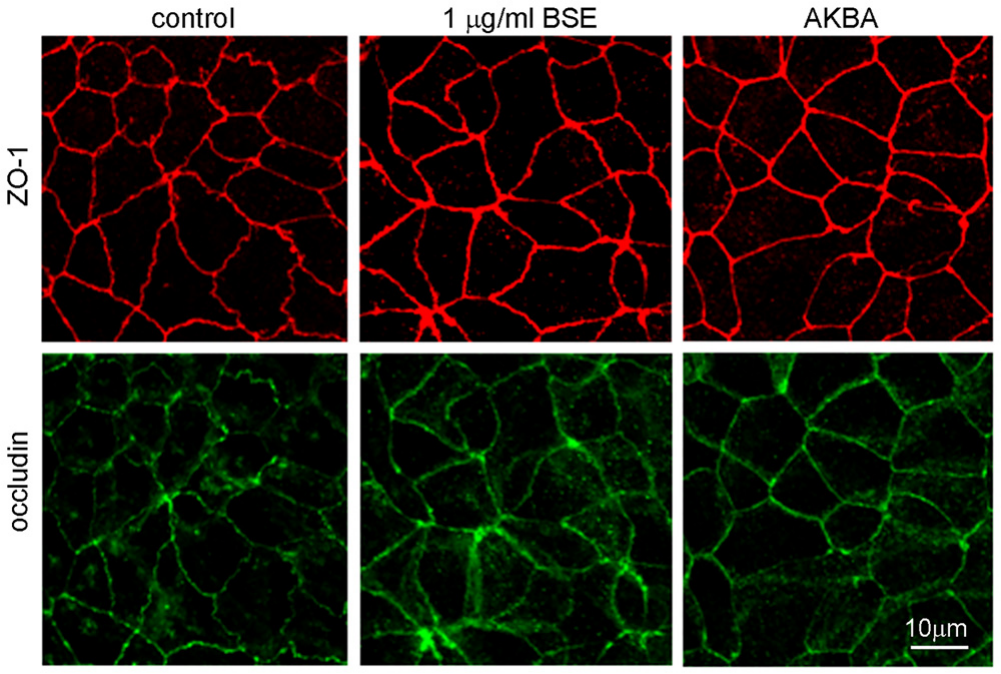
BSE and AKBA effect on occludin and zonula occludens (ZO1) TJ proteins in Caco-2 cell monolayers. BSE and AKBA did not affect basal fluorescent signal of anti-ZO-1 (red) and anti-occludin (green). Images were collected by confocal laser-scanning microscope and are representative of 3 independent experiments. Scale bar = 10 μm.

**Fig 5:**
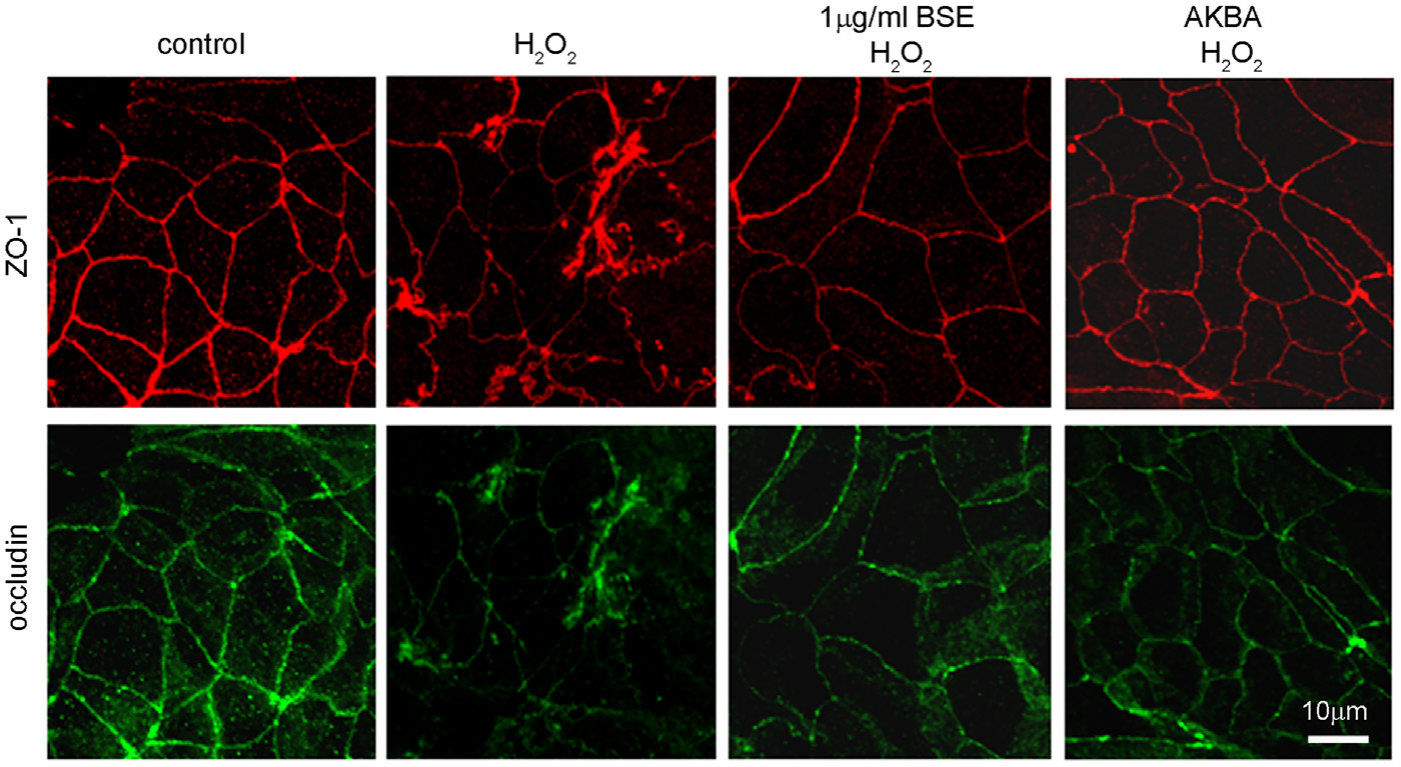
BSE and AKBA ameliorate alterations following exposure to H_2_O_2_ occludin and zonula occludens (ZO1) TJ proteins in Caco-2 cell monolayers. Double immunostaining showing the distribution of ZO-1 (red) and occludin (green). Images were collected by confocal laser-scanning microscope and are representative of 3 independent experiments. Scale bar = 10 μm.

**Fig 6:**
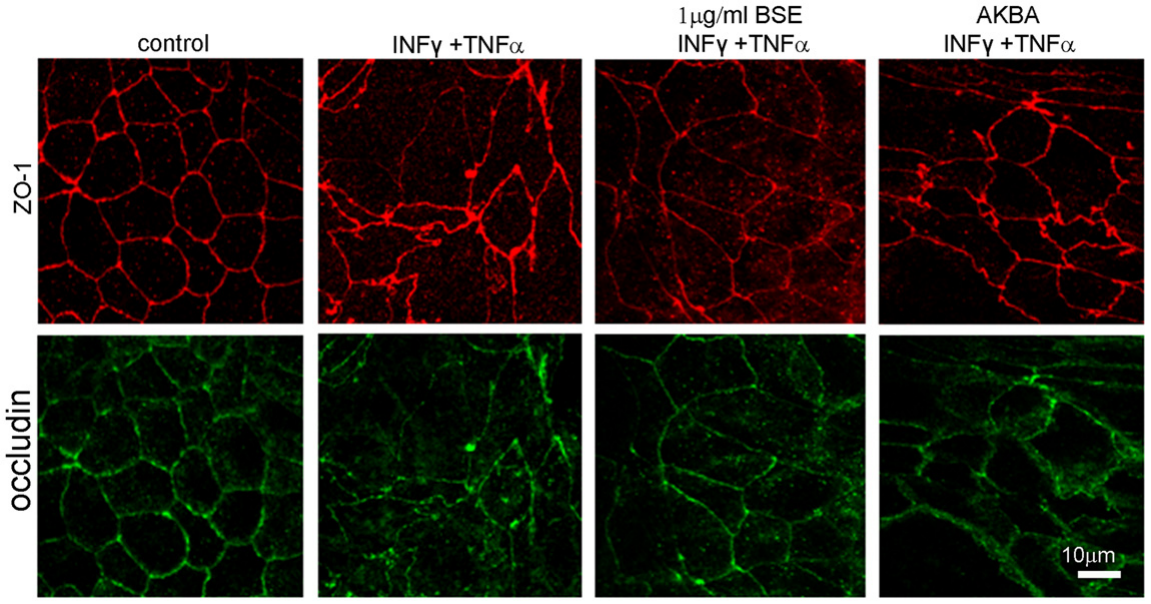
BSE and AKBA ameliorate alterations induced by INF-γ+TNF-α on zonula occludens (ZO1) and occludin TJ proteins in Caco-2 cell monolayers. Double immunostaining showing the distribution of ZO-1 (red) and occludin (green). Images were collected by confocal laser-scanning microscope and are representative of 3 independent experiments. Scale bar = 10 μm.

ZO-1 and occludin belong to TJ proteins that form a continuous, circumferential, belt-like structure at the boundary between the apical and basolateral membrane domains in epithelial and endothelial cells. By constituting a regulated diffusion barrier, TJs establish separate compartments, that are crucial for the exchange of substances through the paracellular pathway, and are considered useful biomarkers of the epithelial barrier function/dysfunction [11, 27]. Therefore the effect of BSE and AKBA on ZO-1 and occludin was studied as possible mechanism involved in protection from inflammatory damage. Results in Fig 4 show that in untreated Caco-2 cell monolayer ZO-1 and occludin immunofluorescence signals localize at the apical membrane junctions, appearing as continuous belt-like structures encircling the cell. This asset was not modified by BSE or AKBA treatments. By contrast, H_2_O_2_ (Fig 5) or INF-γ+TNF-α (Fig 6) cause similar alteration of TJs. Particularly, in INF-γ+TNF-α treated cells it appears that occludin is striking internalized and that the staining of membrane ring structure is irregular. These alterations in TJ proteins caused by the inflammatory stimuli was prevented by both BSE and AKBA treatments.

### BSE and AKBA prevent NF-κB phosphorylation induced by oxidative/inflammatory stimuli

**Fig 7:**
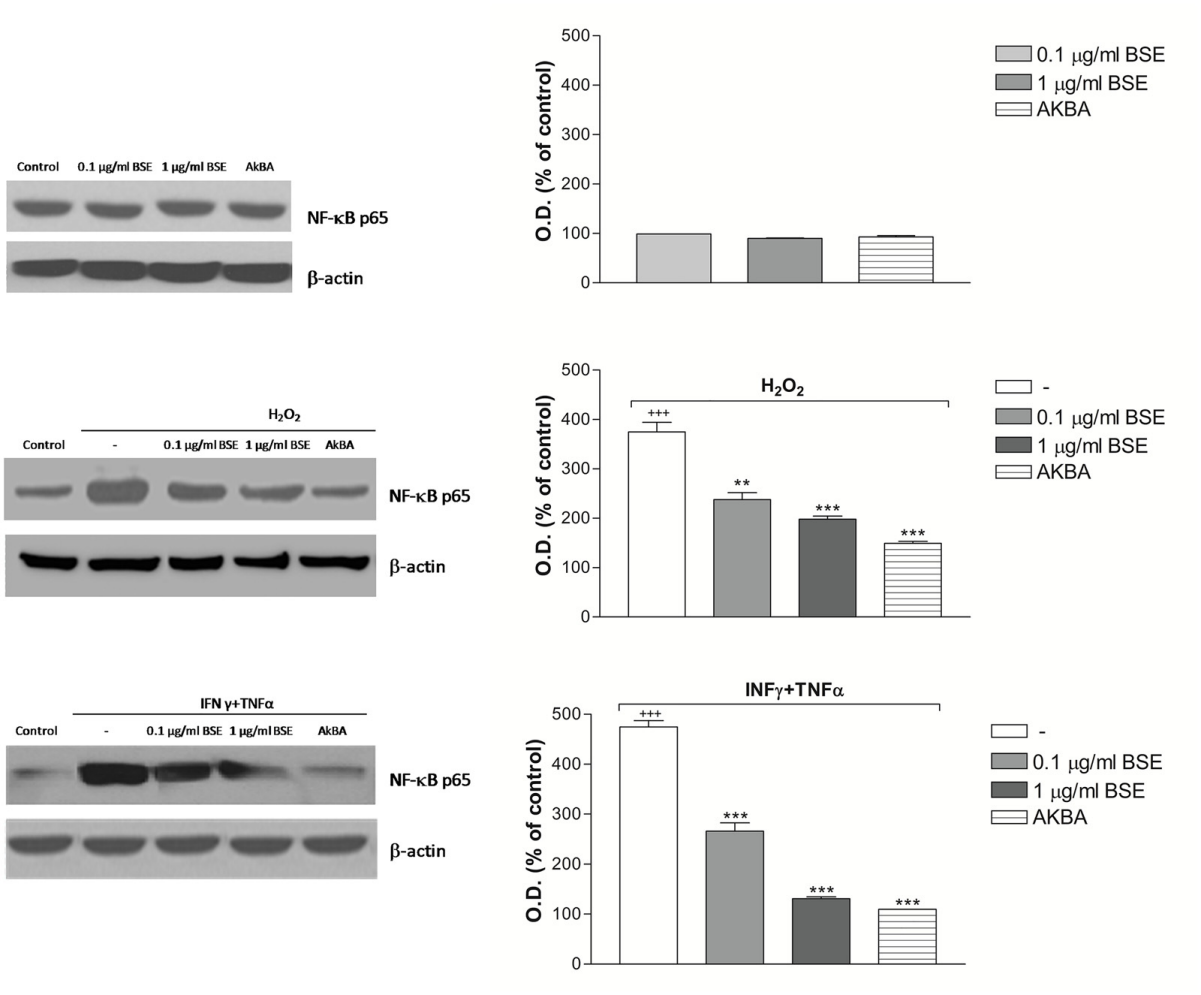
BSE and AKBA effect on NF-kB intracellular level in Caco-2 cells. A) basal condition; B) BSE and AKBA + 4 hours with H_2_O_2_^+++^ *p*<0.001 H_2_O_2_ *vs* control. *** *p*<0.001 BSE and AKBA *vs* H_2_O_2_ ** *p*<0.01 BSE and AKBA *vs* H_2_O_2_. C) 24 hours pretreatment with BSE and AKBA + 4h with INF-γ+TNF-α as described in method. ^+++^ *p*<0.001 INFγ+TNFα *vs* control ^+^ *p*<0.05 INFγ+TNFα *vs* control ***p<0.001 BSE and AKBA *vs* INF-γ+TNF-α. Data are shown as mean ± SEM percentage of control optical density (OD) of n = 3 experiments.

The nuclear transcription factor NF-κB is a key player in the development and progression of chronic inflammatory diseases by regulating a wide range of cytokines such as TNF-α and IL-1β. In IBD patients, NF-κB is markedly induced, strongly influencing the course of mucosal inflammation [28]. To gain further insight into the molecular mechanism of BSE and AKBA, their effect on NF-κB intracellular levels was investigated in Caco-2 cell monolayers after 24 hours of exposure to inflammatory condition (H_2_O_2_ or INFγ + TNFα). Fig 7 shows that BSE and AKBA *per se* did not alter the NF-κB phosphorylation level, whereas, as expected, H_2_O_2_ and INF-γ+TNF-α treatment caused a significant increase of it. Interestingly, pre-treatment with BSE and AKBA positively and significantly counteracted this event.

### BSE and AKBA counteract the ROS generation induced by H_2_O_2_

**Fig 8:**
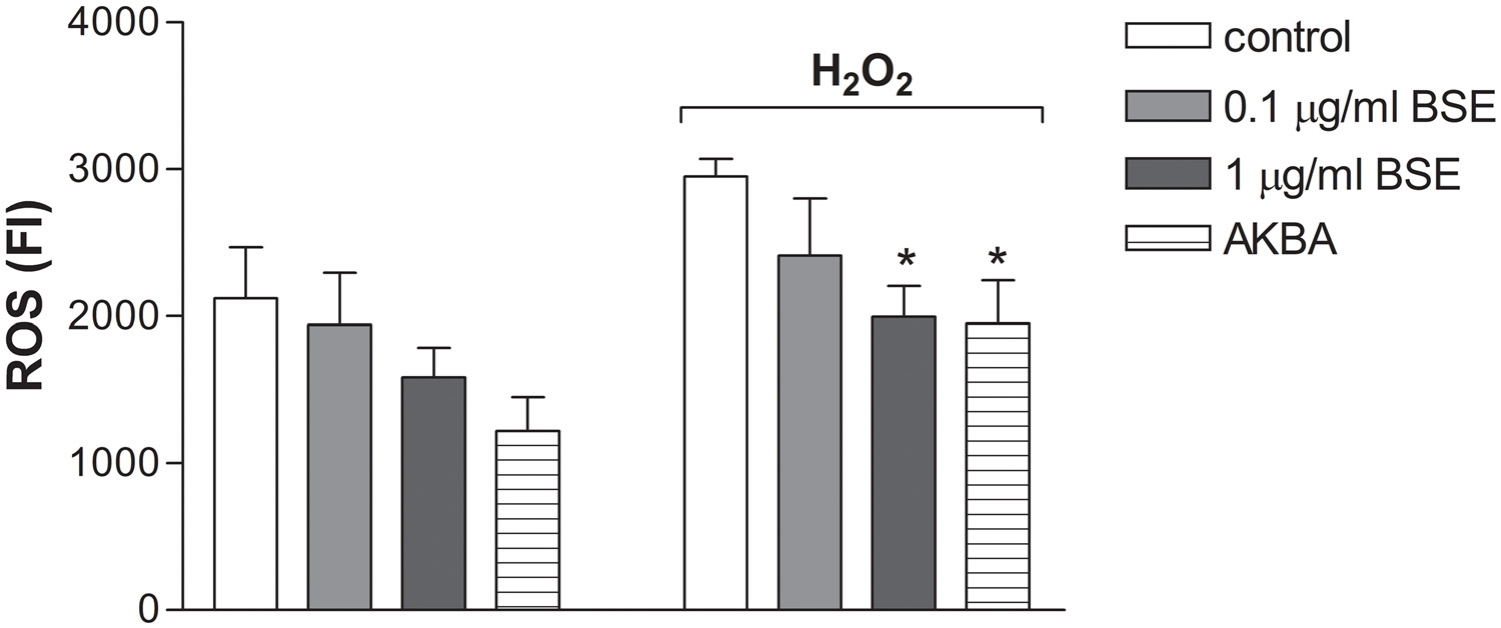
Effect of BSE and AKBA on ROS level in Caco-2 cells. **ROS were measured after 24 hours of incubation in untreated cells and after 500 μM H_2_O_2_ exposure.** Data are expressed as mean ± SEM percentage of basal (100%) DCF fluorescence intensity (FI) of n = 4 experiments. *p<0.05 BSE or AKBA vs H_2_O_2_.

Abnormally high levels of ROS are produced in IBD and their destructive effects may contribute to the initiation and/or propagation of tissue damage [10, 29]. The antioxidant activity of BSE and AKBA was assayed in Caco-2 cell monolayers immediately after the exposure to H_2_O_2_ stimuli. Results in Fig 8 show that BSE and AKBA not only reduced the basal intracellular ROS levels, even if not significantly, but also they significantly counteracted the increased ROS generation induced by the oxidative stimulus (*p* = 0.05).

## Discussion

In Caco-2 cell monolayers BSE and its pure derivative AKBA are able to preserve the intestinal barrier integrity and function from damages caused by H_2_O_2_ or INF-γ+TNF-α inflammatory stimuli. Comparing the quality and the potency of their pharmacological effects it can be assumed that the activity of BSE is mainly due to AKBA.

The inflammation is crucial in gut barrier dysruption in IBD [29–32] and despite *Boswellia serrata* is included among several natural anti-inflammatory compounds, its efficacy in IBD is still debated, stimulating further investigation on its mechanism of action [17, 33, 34].

The *Boswellia serrata* gum extract used in this study was profiled by HPLC-MS and HPLC-DAD-ELSD procedures to determine the triterpene content. The results showed a nearly 34% amount of pentacyclic triterpenes, and AKBA is the compound present at 2.71.%, according to the literature [35]. The safety of BSE and AKBA was demonstrated by unaltered cell viability, cell polarity, paracellular permeability and TJs, biomarkers of intestinal barrier function and integrity. Thereafter Caco-2 cells exposed to INF-γ+TNF-α or H_2_O_2_ were chosen as convenient experimental paradigm of intestinal inflammation, for specific reasons. Caco-2 immortalized cells are an invaluable reductionist model for studying the epithelial barrier and TJ integrity, resembling many of the morphologic and functional attributes of the intestinal epithelium [36–38]. Pro-inflammatory cytokines and ROS contribute to the initiation and/or propagation of damage within the mucosal intestinal barrier in IBD, and they may be used to reproduce in cell cultures comparable endogenous inflammation [10, 29, 39, 40]. Indeed, when we exposed the Caco-2 cell monolayer to INF-γ+TNF-α or H_2_O_2_ results showed significant decrease of the cell polarity (TEER) and increase of the paracellular permeability. Interestingly, these alterations were significantly prevented by pre-treatment with BSE or AKBA.

In human gut a single layer of epithelial cells separates the intestinal lumen from the underlying lamina propria, and the space between these cells is sealed by TJ proteins, such as occludin, ZO-1 and claudins [11, 27, 41]. TJs are essential to maintain physiologic processes in all organs containing epithelia and critical in the intestinal barrier, where they modulate the cell polarity, proliferation, and differentiation [42]. The occludin and ZO-1 delocalization from the TJs is associated with intestinal barrier dysfunction and increased permeability [27, 43]. Various stimuli, including pathogens, oxidative stress, and pro-inflammatory cytokines can affect TJs proteins [11,39], such as TNF-α and IFN-γ re-distribution processes inducing in cell culture and in animal models comparable barrier defects as observed in IBD [40, 44, 45]. According to the literature, our results demonstrated that INF-γ+TNF-α as well H_2_O_2_ caused occludin and ZO-1 disruption on Caco-2 cell membrane and BSE or AKBA efficaciously prevented TJs disassembly.

It has been shown that the increase of intestinal TJ permeability as well as other TNF-α -induced genes are NF-κB-dependent, the relevant transcription factor in inflammatory process [46, 47]. In our experiments INF-γ+TNF-α and H_2_O_2_ exposure caused NF-κB increase in Caco-2 cell monolayers and this activation was significantly counteracted by BSE and AKBA, indicating the possibility of the natural compounds to down-regulate the intestinal response to chronic inflammatory stimuli. Finally, at the same concentration that ameliorates the intestinal damage induced by inflammatory stimuli, BSE and AKBA prevented the ROS intracellular increase induced following exposure to H_2_O_2_. Our results *in vitro* are in accordance with reduced lipid peroxidation, nitric oxide and iNOS as recently shown by *Boswellia serrata* in acute experimental ulcerative colitis experiments [48]. It is known that intact intestinal epithelial barrier but also endogenous antioxidant defence prevent pathogens and antigenic molecules from coming into the mucosa and contacting the immune system; however in some tissue, such as colon, the antioxidant capacity is low and it may facilitate inflammatory injury [49]. Therefore, it can be supposed that this low endogenous antioxidant defence might be overcome by *Boswellia*, thus balancing the several mechanisms involved in the intestinal maintenance and preservation from inflammatory damage.

Kiela *et al*. [50] observed no benefit to disease severity in both the dextran sulphate sodium- or trinitrobenzene sulfonic acid-induced models of colitis where mice were fed with a diet enriched with either hexane or methanolic *Boswellia* extracts. These authors also reported that boswellic acids (50 μM) increased basal and IL-1β-stimulated NF-κB activity in Caco-2 cells transiently transfected with NF-κB reporter plasmid, and considered this effect possibly associated to oxidative stress. However, some differences characterize our *in vitro* model and experimental design from the aforementioned report. In our study, different in vitro inflammatory model and lower concentrations of boswellic acids were used as well as a longer time of exposure compared with the study by Kiela *et al*. [50]. Whether these differences in the treatment with BSE and AKBA account for the discordant anti-inflammatory responses to these agents remains unclear but underline the relevance of specific cellular/tissue antioxidant state and the need for a systematic evaluation of the influence of these factors on therapeutic efficacy of *Boswellia serrata* gum extract in different models of *in vitro* and *in vivo* intestinal inflammation. Indeed, in the last few years it has become evident that persistent dysregulation of redox status contributes to the intensity and duration of the inflammatory response and is involved in the pathogenesis of chronic disease states; therefore the identification of natural compounds as regulators of inflammatory signalling is critical in the discovery of novel therapeutics [51]. Therefore, targeting the re-establishment of intestinal barrier function is still a challenge in acute or chronic enteropathies and the specific functional, morphological and molecular mechanisms in colonic cell lines of *Boswellia* herein demonstrated in Caco-2 cell lines, support its use as safe adjuvant in IBD therapeutic strategies.
